# *Candida* bloodstream infection among children hospitalised in three public-sector hospitals in the Metro West region of Cape Town, South Africa

**DOI:** 10.1186/s12879-023-08027-z

**Published:** 2023-02-03

**Authors:** Mulugeta Naizgi Gebremicael, James J. C. Nuttall, Hafsah D. Tootla, Amanda Khumalo, Lloyd Tooke, Shamiel Salie, Rudzani Muloiwa, Natasha Rhoda, Wisdom Basera, Brian S. Eley

**Affiliations:** 1 Paediatric Infectious Diseases Unit, Department of Paediatrics and Child Health, Red Cross War Memorial Children’s Hospital, University of Cape Town, Cape Town, South Africa; 2 Present Address: Ayder Comprehensive Specialised Hospital, College of Health Sciences, Mekelle University, Mekelle, Ethiopia; 3 Division of Medical Microbiology, National Health Laboratory Service, Red Cross War Memorial Children’s Hospital and Groote Schuur Hospital, University of Cape Town, Cape Town, South Africa; 4 Division of Neonatal Medicine, Department of Paediatrics and Child Health, Groote Schuur Hospital, University of Cape Town, Cape Town, South Africa; 5 Paediatric Critical Care Unit, Department of Paediatrics and Child Health, Red Cross War Memorial Children’s Hospital, University of Cape Town, Cape Town, South Africa; 6 Department of Paediatrics and Child Health, University of Cape Town, Cape Town, South Africa; 7 Division of Neonatal Medicine, Department of Paediatrics and Child Health, Mowbray Maternity Hospital, University of Cape Town, Cape Town, South Africa; 8 School of Public Health and Family Medicine, University of Cape Town, Cape Town, South Africa; 9 Burden of Disease Research Unit, South African Medical Research Council, Cape Town, South Africa

**Keywords:** *Candida* bloodstream infection, Children, Candidaemia, *Candida* species, Antifungal susceptibility, Mortality

## Abstract

**Background:**

*Candida* bloodstream infection (BSI) causes appreciable mortality in neonates and children. There are few studies describing the epidemiology of *Candida* BSI in children living in sub-Saharan Africa.

**Methods:**

A retrospective descriptive study was conducted at three public sector hospitals in Cape Town, South Africa. Demographic and clinical details, antifungal management and patient outcome data were obtained by medical record review. *Candida* species distribution and antifungal susceptibility testing results were obtained from the National Health Laboratory Service database.

**Results:**

Of the 97 *Candida* BSI episodes identified during a five-year period, 48/97 (49%) were *Candida albicans (C. albicans)*, and 49/97 (51%) were non-*C. albicans* species. The overall incidence risk was 0.8 *Candida* BSI episodes per 1000 admissions at Red Cross War Memorial Children’s Hospital. Of the 77/97 (79%) *Candida* BSI episodes with available clinical information, the median age (interquartile range) at the time of BSI was 7 (1–25) months, 36/77 (47%) were associated with moderate or severe underweight-for-age and vasopressor therapy was administered to 22/77 (29%) study participants. Most of the *Candida* BSI episodes were healthcare-associated infections, 63/77 (82%). Fluconazole resistance was documented among 17%, 0% and 0% of *C. parapsilosis, C. tropicalis* and *C. albicans* isolates, respectively. All *Candida* isolates tested were susceptible to amphotericin B and the echinocandins. The mortality rate within 30 days of *Candida* BSI diagnosis was 13/75 (17%). On multivariable analysis, factors associated with mortality within 30 days of *Candida* BSI diagnosis included vasopressor therapy requirement during *Candida* BSI, adjusted Odds ratio (aOR) 53 (95% confidence interval 2–1029); hepatic dysfunction, aOR 13 (95% CI 1–146); and concomitant bacterial BSI, aOR 10 (95% CI 2–60).

**Conclusion:**

The study adds to the limited number of studies describing paediatric *Candida* BSI in sub-Saharan Africa. Non-*C. Albicans* BSI episodes occurred more frequently than *C. albicans* episodes, and vasopressor therapy requirement, hepatic dysfunction and concomitant bacterial BSI were associated with an increase in 30-day mortality.

**Supplementary Information:**

The online version contains supplementary material available at 10.1186/s12879-023-08027-z.

## Background

The reported candidaemia incidence in paediatric patients ranges between 0.2 and 10.5 cases per 1000 admissions [1, 2]. In multicentre, laboratory-based surveillance among hospitalized children in South Africa from 2012 to 2017, the overall incidence risk of paediatric candidaemia at tertiary-level, public-sector hospitals was 5.3 cases per 1000 admissions and ranged from 0.4 to 119.1 per 1000 admissions [3]. A retrospective study on BSI at Red Cross War Memorial Children’s Hospital (RCWMCH), Cape Town, in 2011 and 2012, identified candidaemia in 6.1% of all BSIs [4]. An Egyptian study documented a higher prevalence of 17.3% among all paediatric BSIs [5].

Risk factors for candidaemia in paediatric patients include antibiotic exposure, corticosteroid therapy, the presence of a central venous catheter (CVC), neutropaenia, prior fungal colonisation, and intensive care unit (ICU) admission [6, 7]. Additional risk factors are present during the neonatal period including prematurity, low birth weight (LBW), and related co-morbidities, including total parenteral nutrition (TPN), respiratory disease and mechanical ventilation [6, 8, 9].

Globally, the most prevalent cause of candidaemia has shifted from *Candida albicans (C. albicans)* to non-*C. albicans* spp*.* The frequency of non-*C. albicans* spp. infections varies according to geographical location, patient characteristics and age. In paediatric populations, *C. parapsilosis* is the common non-*C, albicans* sp. associated with candidaemia [10–13]. Recent paediatric studies from Egypt and China reported that non-*C. albicans* spp*.* collectively accounted for the greatest number of cases, although *C. albicans* was the most common species [5, 14]. In contrast, studies from South Africa showed that *C. parapsilosis* was the most prevalent species causing *Candida* BSI followed by *C. albican*s [3, 15]*.*

The shift to non-*C. albicans* candidaemia is associated with reduced susceptibility to fluconazole, a first-line antifungal agent in many settings. Several context-specific reasons may underly this shift such as the selection of non-*C. albicans spp*. under antifungal pressure that are intrinsically resistant to fluconazole e. g. *C. krusei*, or the selection of resistant non-*C. albicans* clones during healthcare-associated outbreaks. In one paediatric study conducted in Turkey, fluconazole resistance was lower among *C. albicans* isolates than non-*C. albicans* isolates, 4.3% versus 16.6%. In this study, the fluconazole-resistant non-*C. albicans* isolates were *C. krusei* isolates [16]. An Egyptian study found that while fluconazole resistance to *C. albicans* and non-*C. albicans* spp. accounted for 38.9% and 44% of the total number of *Candida* spp*.* tested, respectively, susceptibility to caspofungin, amphotericin B and itraconazole was 99%, 97%, and 73%, respectively against all *Candida* isolates tested [5]*. *A neonatal study from Johannesburg reported higher fluconazole resistance among *C. parapsilosis* isolates compared to *C. albicans* isolates. The authors of this study suggested that widespread empiric carbapenem usage due to increasing multiresistant bacterial infection may be driving the selection of fluconazole-resistant *C. parapsilosis* isolates [15]. Similarly, in a South African multicentre study, 35% of all *Candida* isolates were resistant to fluconazole. Fluconazole resistance was higher among *C. parapsilosis* isolates than other *Candida* spp. By contrast, only three of 3061 isolates tested were resistant to echinocandins and all *Candida* isolates were susceptible to amphotericin B [3].

High mortality has been associated with *Candida* BSI. A South African study of neonates with fungal BSI (97% of the 59 episodes were caused by *Candida spp.*) recorded an overall mortality rate of 45.8%. In that study death was significantly associated with LBW and necrotizing enterocolitis [15]. An Egyptian study reported mortality of 64% among children diagnosed with *Candida* BSI, most deaths were associated with non-*C. albicans* BSI [5]. As with previous studies, a recent South African study showed that the overall crude 30-day inpatient mortality for patients with *Candida* BSI is high (38%) and even higher among neonates (43%) and adolescents (43%) [3]. In the Turkish study, treatment in an ICU, the presence of an indwelling CVC, failure to remove an indwelling CVC, and mechanical ventilation during invasive *Candida* infection were associated with mortality [16].

A limited number of studies have described the epidemiology of candidaemia in children in sub-Saharan Africa. The results of a large multicentre laboratory-based surveillance study of culture-confirmed candidaemia in children in South Africa were recently published. Sixty-four percent of the isolates included in that study were from Gauteng province. Furthermore, risk factor analysis for 30-day mortality among neonates with candidaemia, showed that *C. parapsilosis* BSI was associated with lower mortality [3]. The present study was undertaken to describe the recent burden of *Candida* BSI, the clinical presentation, species distribution, antifungal susceptibility, and outcome of *Candida* BSI among children less than 15 years of age admitted to three public sector hospitals in Cape Town, providing contemporary information about this important infection in the Western Cape province of South Africa.

## Methods

### Study design, setting and inclusion criteria

This retrospective descriptive study was conducted at RCWMCH, Groote Schuur Hospital (GSH), and Mowbray Maternity Hospital (MMH). The RCWMCH serves as a tertiary referral centre for children in the Western Cape province and surrounding provinces. GSH is a major tertiary referral centre for adult patients but also provided tertiary neonatal and secondary paediatric inpatient services during the study period. MMH is a dedicated maternal and neonatal regional hospital with limited tertiary service. The study was done in children with culture-confirmed *Candida* BSI, diagnosed between 1 January 2015 and 31 December 2019. All *Candida* BSI episodes diagnosed at RCWMCH were used in the incidence risk calculations for that hospital. Study participants with available clinical records were used to complete the clinical and microbiology descriptions.

### Data collection

The National Health Laboratory Service (NHLS) Central Data Warehouse (CDW) extracted a line list of children admitted to RCWMCH, GSH and MMH with culture-confirmed *Candida* BSI for the period January 2015 to December 2019. This line list included species identification and antifungal susceptibility testing results.

Where available, paper-based medical records of patients with *Candida* BSI episodes were reviewed at RCWMCH, GSH and MMH, and relevant data extracted and manually transferred to standardised data collection forms. The microbiology results obtained from the NHLS CDW were added to the data collection forms.

### Microbiological procedures

Blood culture specimens from the three hospitals were sent to the GSH NHLS microbiology laboratory for processing. The laboratory uses the BacT/ALERT automated blood culture system (BioMérieux, Marcy-l’Etoile, France). Signal-positive blood culture broth was Gram-stained. Broth with yeasts observed using light microscopy, was inoculated onto 2% horse blood agar and Sabouraud Dextrose agar and incubated aerobically at 37 °C. Yeasts cultured between 24 and 48 h were identified with susceptibility testing performed using the Vitek 2 automated system (BioMérieux, Marcy-l’Etoile, France) YST identification and AST-YS08 cards, respectively. Susceptibility test results were interpreted according to published Clinical and Laboratory Standards Institute guidelines [17].

### Study definitions

*Candida* BSI was defined as isolation of any *Candida* spp. from blood culture either collected peripherally or via a central venous catheter (CVC).

*Candida* BSI was classified as (1) infection present on admission (IPOA) if the *Candida* sp. was cultured from a blood culture obtained on the day of admission (calendar day 1), 2 days before admission or the calendar day after admission (calendar day 2), or (2) healthcare-associated infection (HAI) if the *Candida* sp. was isolated from a blood culture obtained on or after the 3^rd^ calendar day of admission [18].

Pre-term birth: a gestational age (GA) < 37 completed weeks at birth.

Concomitant bacteraemia: Isolation of a bacterial isolate from the blood culture specimen in which the *Candida* sp. was isolated.

Human immunodeficiency virus (HIV) status was defined as follows: (1) HIV-infected: a child < 18 months old with a positive HIV deoxyribonucleic acid (DNA) polymerase chain reaction (PCR) result confirmed by either a quantitative HIV ribonucleic acid (RNA) PCR or repeat HIV DNA PCR test, or a child ≥ 18 months old with 2 positive serological test results (HIV enzyme-linked immunosorbent assay or HIV Rapid test) or a positive HIV DNA PCR result confirmed by either a quantitative HIV RNA PCR or repeat HIV DNA PCR test, (2) HIV-uninfected: a child with a negative HIV serological or HIV DNA PCR result and (3) unknown HIV status: a child with unknown maternal HIV status and who was not tested for HIV infection [19].

Moderate and severe underweight for age (UWFA) were defined as weight-for-age z score (WAZ) between − 2 and − 3 standard deviations (SD) of the World Health Organisation (WHO) growth reference standards, and a WAZ <  − 3 SD, respectively [20].

The urinary tract was regarded as the site of infection if one of the following criteria was fulfilled: a urine culture yielding a single organism with > 1000 colony-forming units/mL from a suprapubic aspiration or > 10,000 colony-forming units/mL via urethral catheterization [21].

Disseminated intravascular coagulopathy (DIC)*:* A prothrombin time of ≥ 2 s, an activated partial thromboplastin time of ≥ 60 s or a fibrinogen level of < 2 μmol/L [22].

Renal dysfunction*:* a serum creatinine concentration above the normal age-related range [23, 24].

Liver dysfunction*:* a ≥ twofold increase of serum aspartate aminotransferase and/or serum alanine aminotransferase concentration and/or a total bilirubin in a child more than 28 days old of > 70 μmol/L [24, 25].

### Statistical analysis

The data were analysed using STATA/IC version 14.2 (College Stata, TX, USA). The incidence risk of *Candida* BSI was calculated per 1000 hospital admissions for RCWMCH. Continuous variables were expressed as medians (interquartile range, IQR) since the continuous data were skewed. Proportions and percentages were used to describe categorical variables.

The Wilcoxon rank-sum test for independent variables was used to compare the continuous data stratified by type of *Candida* BSI. The association between categorical variables was done using Pearson’s Chi-square test (χ^2^) or Fisher’s Exact test as appropriate. Two-tailed p-values ≤ 0.05 were considered statistically significant.

Univariable and multivariable logistic regression were used to identify factors independently associated with mortality within 30 days of the date of the blood culture from which *Candida* sp*.* was isolated. The multivariable logistic regression model was built by incorporating all variables utilised in the univariable analysis. The multivariable logistic regression model results were expressed as adjusted odds ratios (aORs) and 95% confidence intervals (95% CIs). Mortality associated with *C*. *albicans* versus non-*C. albicans* BSI was analysed using Kaplan–Meier survival estimates and compared between the two using the log rank test.

## Results

### Study participants

**Fig. 1:**
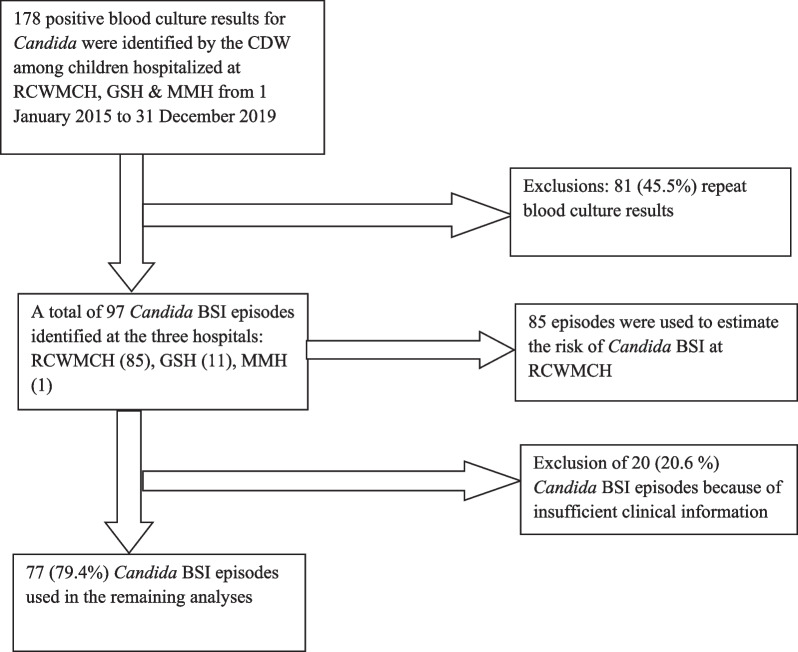
Selection of *Candida* bloodstream infection episodes for data analysis. *CDW* Central Data Warehouse, *Candida* BSI *Candida* bloodstream infection, *RCWMCH* Red Cross War Memorial Children’s Hospital, *GSH* Groote Schuur Hospital, *MMH* Mowbray Maternity Hospital

During the study period, there were 97 *Candida* BSI episodes in 97 participants, 85 episodes at RCWMCH, 11 at GSH and one at MMH. The 85 *Candida* BSI episodes at RCWMCH were used to estimate the risk of *Candida* BSI at that hospital. There was insufficient clinical information on twenty episodes of *Candida* BSI thus 77 (79%) *Candida* BSI episodes were used in all subsequent analyses (Fig. 1).

### Risk of *Candida* bloodstream infection at RCWMCH

**Fig. 2:**
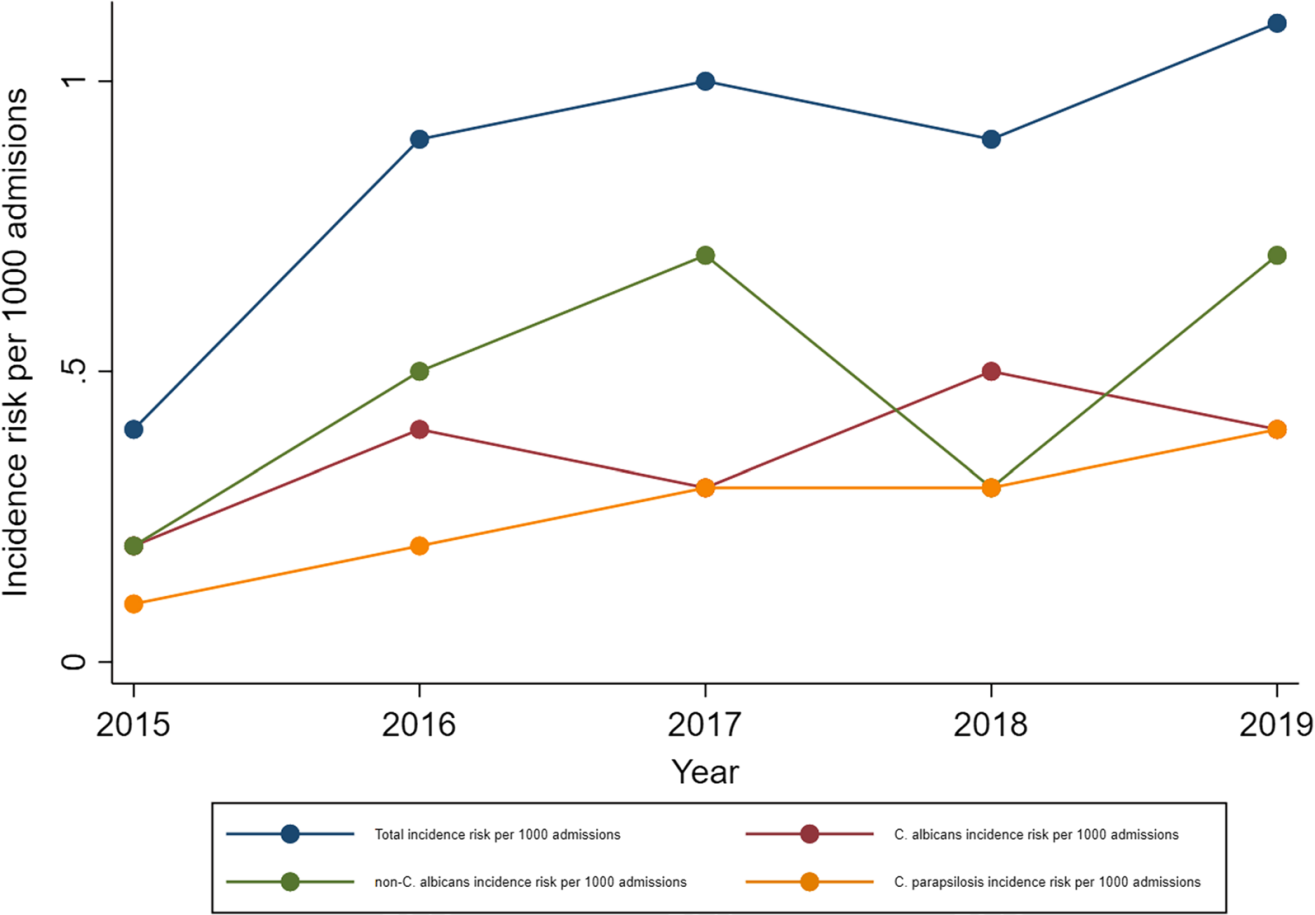
Annual change in incidence risk per 1000 hospital admissions of *Candida* bloodstream infection episodes at Red Cross War Memorial Children’s Hospital, 2015–2019

The overall incidence risk throughout the study period was 0.8 episodes/1000 hospital admissions for all *Candida* BSI episodes, 0.4 episodes/1000 hospital admissions for all *C. albicans* BSI episodes, 0.5 episodes/1000 hospital admissions for all non-*C. albicans* BSI episodes, and 0.3 episodes/1000 hospital admissions for all *C. parapsilosis* BSI episodes; *C. parapsilosis* being the main contributor to *non-C. albicans* BSI episodes. With exception of 2018, the overall annual incidence risk increased progressively throughout the study period (Fig. 2). This increase as well as the decline in 2018 were mainly attributed to changes in the incidence risk of non-*C. albicans* over time (Fig. 2; Additional file 1: Table S1).

### Baseline characteristics

Table 1 describes demographic and clinical characteristics of 77 *Candida* BSI episodes in 77 study participants. The median age at the time of *Candida* BSI was 6.8 months, IQR 1–25. Close to 50% of the episodes occurred in participants who were moderately or severely underweight for age. Of the 52 participants (68%) whose HIV status was known at the time of their C*andida* BSI events, only two were HIV-infected. The proportion of participants who were admitted in ICU during the *Candida* BSI was higher among those with *C. albicans* BSI episodes compared to those with non-*C. albicans* BSI episodes, 79% (30/38) vs. 49% (19/39), p = 0.01. Furthermore, a higher proportion of non-*C. albicans* than *C. albicans* BSI episodes occurred in participants with haematological conditions or cancer on immunosuppressive treatment 28% (11/39) vs*.* 5% (2/38), p = 0.01.

**Table 1:** Characteristics of study participants at the time of *Candida* bloodstream infection

	Total N = 77^a^	*C. albicans* BSI N = 38^a^	Non-*C. albicans* BSI N = 39^a^	P value**
Median age (IQR) in months	7 (1–24)	5 (1–19)	9 (2–58)	0.2
Age category				
< 1 month 1—12 months 1—5 years > 5 years	14 (18) 29 (38) 21 (27) 13 (17)	8 (21) 15 (39) 11 (29) 4 (11)	6 (15) 14 (36) 10 (26) 9 (23)	0.5
Sex				
Male Female	38 (49) 39 (51)	21 (55) 17 (45)	17 (44) 22 (56)	0.3
HIV status				
HIV-infected HIV-uninfected Unknown	2 (3) 50 (65) 25 (32)	2 (5) 22 (58) 14 (37)	0 (0) 28 (72) 11 (28)	0.2
Weight-for-age, Z score category				
Normal weight Moderate underweight Severe underweight	41 (53) 6 (8) 30 (39)	19 (50) 2 (5) 17 (45)	22 (57) 4 (10) 13 (33)	0.5
Gestational age of infants (< 12 months of age) at birth				
Term Preterm Unknown	13/43 (30) 24/43 (56) 6/43 (14)	4/23 (17) 14/23 (61) 5/23 (22)	9/20 (45) 10/20 (50) 1/20 (5)	0.08
Recorded temperature (°C)				
< 35.5 35.5–37.9 ≥ 38.0	1/66 (2) 29/66 (44) 36/66 (54)	0/31 (0) 17/31(55) 14/31 (45)	1/35 (3) 12/35 (34) 22/35 (63)	0.1
Exposure to selected IV antibiotics in the preceding 12 months^b^	63 (82)	33 (87)	30 (77)	0.3
Corticosteroid therapy for more than a month	9 (12)	2 (5)	7 (18)	0.2
Presence of central venous or arterial catheter	57 (74)	30 (79)	27 (69)	0.3
Total parental nutrition	25 (33)	13 (34)	12 (31)	0.7
Necrotizing enterocolitis	11/43 (26)	8/23 (35)	3/20 (15)	0.1
Haematological/cancer patients on immunosuppressive treatment	13 (17)	2 (5)	11 (28)	***0.01***
Haemodialysis	2 (3)	2 (5)	0 (0)	0.2
Present in ICU at the time of *Candida* BSI	49 (64)	30 (79)	19 (49)	***0.01***
Abdominal surgery during the previous 3 months	21 (27)	11 (29)	10 (26)	0.7
Cardiac surgery during the previous 3 months	7 (9)	1 (3)	6 (15)	0.1

Proportions reported as n/N (%)

p-values for categorical variables calculated using Pearson’s Chi-square test or Fishers exact test and those for continuous variables calculated using Wilcoxon rank-sum test for independent variables

*C. albicans Candida albicans*, non-*C. albicans* non-*Candida albicans*, *BSI* bloodstream infection, °C degrees Celsius, *ICU* intensive care unit, *IV* intravenous

^a^N denominator used unless otherwise stated

^b^Carbapenems, vancomycin, aminoglycosides, penicillin, cephalosporins, piperacillin tazobactam, fluoroquinolones and clindamycin

**Comparison of *C. albicans* and non-*C*. *albicans* BSI

### Classification and clinical manifestations

Of the 77 *Candida* BSI episodes, 63 (82%) episodes were HAIs and 14 (18%) were IPOA. The median time between admission and the development of *Candida* BSI in study participants who experienced HAI was 12 days, IQR 7.0–21.0. A clinical site of infection was identified in 44% of the *Candida* BSI episodes. Urinary tract as a focus of infection (37% vs. 3%) and renal dysfunction (37% vs. 23%) as a complication were more common in *C. albicans* BSI than non-*C. albicans* BSI, p < 0.001. Vasopressor therapy was administered in 22 (29%) episodes and concomitant bacterial BSI was documented in 30% of episodes (Table 2).

**Table 2:** Classification, site of infection and complications of *Candida* bloodstream infection

	Total N = 77	*C. albicans* BSI N = 38	Non-*C. albicans* BSI N = 39	P value*
Classification of *Candida* bloodstream infection				
Infection present on admission	14 (18)	6 (16)	8 (21)	0.8
Healthcare-associated infection	63 (82)	32 (84)	31 (80)	0.8
Site of infection				
No definable focus of infection	43 (56)	17 (45)	26 (66)	0.07
Urinary tract	15 (20)	14 (37)	1 (3)	** < *0.001***
Intravenous/arterial catheter	17 (22)	5 (13)	12 (31)	0.1
Abdomen	2 (3)	2 (5)	0 (0)	0.2
Complications				
Disseminated intravascular coagulopathy	9 (12)	3 (8)	6 (15)	0.5
Vasopressor therapy requirement	22 (29)	15 (40)	7 (18)	0.05
Renal dysfunction	15 (20)	14 (37)	1 (3)	** < *0.001***
Confusion	35 (46)	20 (53)	15 (39)	0.3
Liver dysfunction	15 (20)	7 (18)	8 (21)	1.0
Co-infection				
Concomitant bacterial BSI	23 (30)	12 (32)	11 (28)	0.8

*C. albicans* *Candida albicans*, non-*C. albicans* non-*Candida albicans*, *BSI* bloodstream infection

*Comparison of *C. albicans* and non-C. *albicans* BSI

Additional file 2: Table S2 summarises the pathogens causing concomitant bacterial BSI and their antibiotic susceptible patterns

### Species distribution and antifungal susceptibility of the *Candida* isolates

During the study period, a total of 77 *Candida* specie*s* were isolated from the 77 children with complete and available clinical information. Of these, 38 (49%) were *C. albicans* and all were susceptible to fluconazole. Among the *non-C. albicans* isolates, the most frequent species isolated were *C. parapsilosis* (31%), *C. tropicalis* (8%), *and C. glabrata* (5%). Fluconazole resistance was documented in 13% of *C. parapsilosis* isolates and 4% categorised as susceptible-dose dependant*.* All *Candida* isolates tested were susceptible to amphotericin B and the echinocandins (Table 3).

**Table 3:** Distribution and antifungal susceptibility results of *Candida* species causing *Candida* bloodstream infection

*Candida species*	Antifungal susceptibility results				
		Fluconazole	Amphotericin B	Echinocandins	Voriconazole
*C. albicans* (n = 38)	No. isolates tested	38	38	35	38
	No. (%) Susceptible	38(100)	38 (100)	35 (100)	38 (100)
*C. parapsilosis* (n = 24)	No. isolates tested	24	24	23	24
	No. (%) Susceptible	20 (83)^d^	24 (100)	23 (100)	22 (92)^e^
*C. glabrata*^a^ (n = 4)	No. isolates tested	2	4	3	0
	No. (%) Susceptible	–	4 (100)	3 (100)	–
*C. tropicalis* (n = 6)	No. isolates tested	6	6	6	6
	No. (%) Susceptible	6 (100)	6 (100)	6 (100)	6 (100)
*C. krusei*^b^ (n = 3)	No. isolates tested	3	3	2	3
	No. (%) Susceptible	0 (0)	3 (100)	2 (100)	3 (100)
*C. lusitaniae*^c^ (n = 1)	No. isolates tested	1	1	1	0
	No. (%) Susceptible	–	–	–	–
*C. magnolae*^c^ (n = 1)	No. isolates tested	0	0	0	0
	No. (%) Susceptible	–	–	–	–

No., number

^a^*C. glabrata*: There is no susceptibility category for fluconazole, or interpretive criteria for voriconazole

^b^*C. krusei*: This organism is assumed to be intrinsically resistant to fluconazole

^c^There are no interpretive criteria for these organisms

^d^Three isolates (13%) were categorised as fluconazole resistant, and one isolate (4%) categorised as susceptible-dose dependent according to Clinical and Laboratory Standards Institute guidelines, refer reference 17

^e^Two isolates (8%) were categorised as voriconazole resistant according to Clinical and Laboratory Standards Institute guidelines, refer reference [17]

### Antifungal therapy

Of the 77 BSI episodes, 69 (90%) were treated with an antifungal agent. Of the eight episodes (10%) that were not treated at RCWMCH, MMH or GSH, two patients died, two were transferred to another hospital before the diagnosis of *Candida* BSI was established, and four remained well despite not receiving antifungal therapy. Antifungal therapy was initiated a median of 1 day (IQR, 1–2) after the first diagnostic blood culture was performed. The median duration of all antifungal therapy per episode was 14 days (IQR, 13–20). In 58 of the 69 treated episodes (84%), at least one repeat blood culture was performed during the BSI episode. The median time (IQR) between initial blood culture and repeat blood culture was 2 (2–4) days. The repeat cultures were negative in 56 of these 58 episodes (97%). Thus, a negative blood culture was documented in 56/69 (81%) of the treated BSI episodes. A negative blood culture result was not documented in the two episodes in which single repeat cultures were still positive as no further blood cultures were performed. The median time (IQR) until repeat negative blood culture from initiation of treatment was 4 (2–7) days. The median duration (IQR) of antifungal therapy from negative repeat culture was 13 (10–15) days. After the antifungal susceptibility results became available, the initial antifungal agent was continued in 49 (71%) episodes and changed in 20 (29%) episodes due to resistance (2) or de-escalation (18) to agents with narrower spectrum of antifungal activity. In 5 of the 49 episodes (10%) in which initial antifungal therapy was continued, the susceptibility results indicated that de-escalation was possible but not implemented. Fluconazole was the most frequently used initial antifungal agent, 43/69 (62%), followed by amphotericin B, 21/69 (30%), and caspofungin, 5/69 (7%). After adjustments were made in response to the antifungal susceptibility results, the final treatment regimens were fluconazole, 51/69 (74%), amphotericin B 13/69 (19%) and caspofungin, 5/69 (7%).

### Outcome

**Fig. 3:**
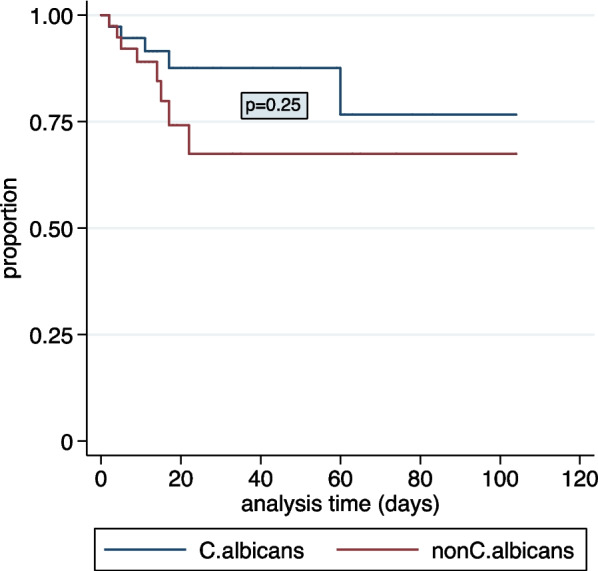
Kaplan–Meier survival estimates for *Candida* bloodstream infection-related mortality

74% (57/77) of *Candida* BSI episodes were successfully treated and the children were discharged from hospital after these episodes. During the study period, 14 (18%) children died during or after the *Candida* BSI episode but prior to hospital discharge (Table 4). Thirteen of these deaths occurred within 30 days of *Candida* BSI diagnosis and were included in the survival analysis. The median time (IQR) to death of these 13 children was 9 (3–16) days. Table 5 describes risk factors associated with mortality within 30 days of *Candida* BSI diagnosis in children with *Candida* BSI. On multivariable analysis, vasopressor therapy requirement, hepatic dysfunction, and concomitant bacterial BSI during *Candida* BSI were significantly associated with mortality within 30 days of *Candida* BSI diagnosis. Furthermore, Kaplan–Meier survival analysis showed that there was no significant difference in the survival of patients with *C. albicans* compared to non-*C. albicans* BSI episodes, p = 0.3 (Fig. 3).

**Table 4:** Outcome associated with *Candida* bloodstream infection

	Total N = 77	*C. albicans* BSI N = 38	Non-*C. albicans* BSI N = 39	P value*
Recovered after antifungal therapy	57 (74)	31 (82)	26 (67)	0.1
Death within 30 days of *Candida* BSI	13 (17)	5 (13)	8 (21)	0.6
Death after 30 days of *Candida* BSI	1 (1)	1 (3)	0 (0)	1.0
Unknown, transferred to another hospital before antifungal therapy initiated	2 (3)	1 (3)	1 (3)	1.0
Not treated with antifungal therapy, remained well	4 (5)	0 (0)	4 (10.)	0.1

*C. albicans* *Candida albicans*, non-*C. albicans* non-*Candida albicans*, *BSI* bloodstream infection

*Comparison of *C. albicans* and non-C. *albicans* BSI

**Table 5:** Risk factors associated with mortality within 30 days of *Candida* bloodstream infection diagnosis

Risk factors for mortality	Patients who died during the follow-up period	Patients who survived	Univariable analysis		Multivariable analysis	
	N = 14	N = 63	OR (95% CI)	p-value	aOR (95% CI)	p-value
Age less than 12 months	9 (64)	35 (56)	1.4 (0.4–4.8)	0.6	0.4 (0.04–3.7)	0.4
Severe underweight (weight for age < -3 z score)	7 (50)	23 (37)	1.7 (0.5–5.6)	0.4	7.4 (0.9–61.5)	0.1
Disseminated intravascular coagulopathy	5 (36)	4 (6)	8.2 (1.8–36.4)	***0.01***	6.9 (0.6–74.8)	0.1
Required vasopressor therapy	9 (64)	13 (21)	6.9 (2.0–24.2)	** < *0.01***	53.3 (2.3–1028.7)	***0.01***
Renal dysfunction	3 (21)	12 (19)	1.2 (0.3–4.8)	0.8	1.2 (0.1–15.3)	0.9
Hepatic dysfunction	6 (43)	9 (14)	4.5 (1.3–16.1)	***0.02***	12.7 (1.1–145.8)	***0.04***
White cell count < 4000 cells/µL	3 (21)	13 (21)	1.2 (0.3–4.8)	0.8	–	–
Presence of an immunosuppressive condition^a^	2 (14)	11 (17)	0.8 (0.2–4.0)	0.8	0.6 (0.03–11.7)	0.7
Present in ICU during *Candida* BSI	10 (71)	39 (62)	1.5 (0.4–5.5)	0.5	0.02 (0–1.3)	0.1
Presence of definable focus of infection	7 (50)	28 (44)	1.3 (0.4–4.0)	0.7	0.9 (0.1–6.3)	0.9
Presence of concomitant bacterial BSI during *Candida* BSI	9 (64)	14 (22)	6.3 (1.8–21.9)	** < *0.01***	9.5 (1.5–60.0)	***0.02***
*Non-Candida albicans* BSI	8 (57)	31 (49)	1.4 (0.4–4.4)	0.6	3.6 (0.3–49.6)	0.3

*BSI* bloodstream infection, *ICU* intensive care unit, *OR* odds ratio, *95% CI* 95% confidence interval, *aOR* adjusted odds ratio

^a^Presence of immunosuppressive condition: study participants with haematological conditions or cancer patients on immunosuppressive treatment, HIV infection, corticosteroid therapy for more than a month

## Discussion

The exact incidence of *Candida* BSI in children in sub-Saharan Africa is not known due to a lack of systematic epidemiological data. In multicentre laboratory-based surveillance among hospitalized children at public-sector hospitals in South Africa, the overall incidence was 5.3 *Candida* BSI episodes per 1000 admissions and ranged from 0.4 to 119.1 per 1000 admissions [3]. The incidence risk in our study of 0.8 per 1000 hospital admissions is consistent with this report, although at the lower end of the range. It is also at the lower end of the range of incidence estimates reported by non-South African studies of 0.2–10.5 *Candida* BSI episodes per 1000 admissions [1, 2]. Good infection prevention practice, less crowded wards, better infrastructure and staffing resources, and presence of antifungal stewardship programmes are possible factors that contribute to lower incidence [26, 27].

Many of the children in our study had interventions and underlying medical conditions that are likely to have predisposed them to *Candida* BSI. These included prior exposure to antibiotics, the presence of CVCs, parenteral nutrition, treatment in the ICU, and malignancy, and in neonates and infants, preterm birth and necrotising enterocolitis. Similar risk factors have been reported in other studies, including the recent South African multicentre laboratory-based surveillance report [3, 6–9].

In our study, non-*C. albicans* spp*.* collectively accounted for the greater number of cases, although *C. albicans* was the most common single species accounting for nearly half of all *Candida* BSI episodes. Similarly, recent paediatric studies from Egypt and China showing a shift to non-*C. albicans*, reported that *C. albicans* was still the predominant species [5, 14]. A multicentre paediatric *Candida* BSI study showed that between 2016 and 2017, *C. parapsilosis* was the most prevalent species in South Africa although *C*. *albicans* remained predominant in less populous provinces [3]. In our study, *C*. *parapsilosis* was the most common non-*albicans Candida* sp. accounting for 31.2% of all *Candida* isolates. A retrospective cohort study showed that in adult haematological patients with neutropaenia, *non-C. albicans* species were detected much more frequently than in non-neutropaenic patients. Furthermore, patients with CVCs in situ were at high risk for *C. parapsilosis* BSI and fluconazole prophylaxis was a risk factor for both *C. glabrata* and *C. krusei* BSI. [28]. Similarly, in our study, more than half (55%) of the haematologic patients with non-*C. albicans* had severe neutropaenia (polymorphonuclear neutrophil count < 500 cells/µL). Additionally, all children in our study who developed *C. parapsilosis* BSI had CVCs, but none with haematological conditions or cancer were receiving fluconazole prophylaxis.

Unlike previous studies, we found that all *C. albicans* and *C. tropicalis* isolates and 83% of *C. parapsilosis* isolates were susceptible to fluconazole [3, 5]. In a South African multicentre study, over half of the *C*. *parapsilosis* isolates were resistant to fluconazole [3]. This contrasts with our finding that only 17% of *C. parapsilosis* isolates were either resistant or susceptible-dose dependant to fluconazole. The lower fluconazole resistance rate might be due to differences in infection prevention and control practices and hospital antifungal stewardship. In our study, all *Candida* isolates tested were susceptible to amphotericin B and echinocandins. This is consistent with a large South African study, in which only three of 3061 *Candida* isolates tested were resistant to echinocandins and all isolates were susceptible to amphotericin B [3]. A study from Egypt published in 2019 also reported very high caspofungin and amphotericin B susceptibilities among *Candida* BSI isolates tested [5].

In our study, mortality within 30 days of *Candida* BSI diagnosis was 17% of 75 patients with known outcomes. This is lower than that reported in an Egyptian study of children with *Candida* BSI (64%), a South African study of neonates with fungal BSI (46%) and a large multicentre study of hospitalised children with *Candida* BSI (38%) [3, 5, 15]. The lower mortality documented in our study may be related to low fluconazole resistance, early initiation of antifungal therapy, the duration of antifungal therapy administered to our patients, and the availability of support services such as ICU care. In the Egyptian study, most deaths were associated with non-*C. albicans* BSI [5]. Although more children who died in our study had non-*C. albicans* BSI compared to those who survived, this difference was not statistically significant.

After adjusting for possible confounding, vasopressor therapy requirement, hepatic dysfunction and concomitant bacterial BSI during *Candida* BSI were significant risk factors associated with mortality within 30 days of *Candida* BSI diagnosis. To our knowledge, previous paediatric studies have not identified concomitant bacterial BSI as a risk factor. However, given that bacterial BSI itself is associated with appreciable mortality, concomitant bacterial BSI is likely to significantly increase the mortality risk during *Candida* BSI as suggested by our findings.

### Study strengths and limitations

The results underline an important antimicrobial stewardship principle, namely that low fluconazole resistance in *Candida* BSI at our institutions implies that fluconazole should still be used for the empiric treatment of *Candida* BSI, thus preserving the echinocandins for the treatment of fluconazole-resistant isolates.

Due to the retrospective study design, bias was unavoidable due to limitations in the completeness and availability of laboratory and clinical data. The gestational ages of preterm infants were not available; thus, it was not possible to determine whether the incidence of *Candida* BSI was higher in extremely premature infants. Matrix assisted laser desorption ionization time of flight mass spectrometry (MALDI-ToF–MS) and reference microdilution methods were not available during the study period, limiting the characterisation of the fungal isolates. Additionally, because of few *Candida* BSI episodes at GSH & MMH our incidence risk calculations were confined to RCWMCH. Furthermore, the sample size was small and underpowered to fully explore risk factors associated with mortality within 30 days of *Candida* BSI diagnosis. Differences between our 30-day post-*Candida* BSI diagnosis mortality rate and higher rates documented in other sub-Saharan African studies suggest that our results may not be generalizable to other hospitals. Despite these limitations, our findings provide important insights into the epidemiology and clinical manifestations of paediatric *Candida* BSI at our institutions.

## Conclusion

Our study provides a description of *Candida* BSI in children in the Western Cape province of South Africa, indicating low incidence, low fluconazole resistance and relatively low mortality. Vasopressor therapy requirement, hepatic dysfunction, and concomitant bacterial BSI were independent risk factors associated with mortality within 30 days of *Candida* BSI diagnosis. Further research, particularly prospective studies, is required to provide a complete understanding of the impact of *Candida* BSI on the childhood population in sub-Saharan Africa, including detailed molecular characterisation of the *Candida* isolates.

## Supplementary Information

**Additional file 1: Table S1. **Annual change in incidence risk per 1000 hospital admissions of *Candida* bloodstream infection episodes at Red Cross War Memorial Children’s Hospital, 2015-2019.

**Additional file 2: Table S2. **Spectrum of bacterial isolates causing concomitant bacterial BSI and their antibiotic susceptibility patterns during *Candida* BSI.

## Data Availability

The datasets used and/or analysed during the current study are available from the corresponding author on reasonable request.

## Abbreviations

aOR: Adjusted odds ratio
BSI: Bloodstream infection
CDW: Central Data Warehouse
CI: Confidence interval
CLSI: Clinical and Laboratory Standards Institute
CVC: Central venous catheter
GSH: Groote Schuur Hospital
GA: Gestational age
HAI: Healthcare-associated infection
HIV: Human Immunodeficiency Virus
HREC: Human Research Ethics Committee
ICU: Intensive care unit
IQR: Interquartile range
IPOA: Infection present on admission
LBW: Low birth weight
MMH: Mowbray Maternity Hospital
NHLS: National Health Laboratory Service
OR: Odds ratio
RCWMCH: Red Cross War Memorial Children’s Hospital
SA: South Africa
SD: Standard deviation
SAJID: Southern African Journal of Infectious Diseases
Sp.: Species (singular tense)
Spp.: Species (pleural tense)
TPN: Total parenteral nutrition
VLBW: Very low birth weight
WAZ: Weight-for-age Z score
WHO: World Health Organization
