# Supplementary material for: Candida bloodstream infection among children hospitalised in three public-sector hospitals in the Metro West region of Cape Town, South Africa
Source: BMC Infect Dis. 2023 Feb 3;23:67. doi: 10.1186/s12879-023-08027-z (PMC9896677; doi:10.1186/s12879-023-08027-z)
Supplement: Supplementary file 1 — Additional file 1: Table S1. Annual change in incidence risk per 1000 hospital admissions of Candida bloodstream infection episodes at Red Cross War Memorial Children’s Hospital, 2015-2019. [file 12879_2023_8027_MOESM1_ESM.doc]

**Table S1:** Annual change in incidence risk per 1000 hospital admissions of *Candida* bloodstream infection episodes at Red Cross War Memorial Children’s Hospital, 2015-2019

| Year | Total incidence per 1000 hospital admissions | *C. albicans* incidence per 1000 hospital admissions | Non-*C. albicans* incidence per 1000 hospital admissions | *C. parapsilosis* incidence per 1000 hospital admissions |
| --- | --- | --- | --- | --- |
| 2015 | 0.4 | 0.2 | 0.2 | 0.1 |
| 2016 | 0.9 | 0.4 | 0.5 | 0.2 |
| 2017 | 1.0 | 0.3 | 0.7 | 0.3 |
| 2018 | 0.9 | 0.5 | 0.3 | 0.3 |
| 2019 | 1.1 | 0.4 | 0.7 | 0.4 |
