# Supplementary material for: Candida bloodstream infection among children hospitalised in three public-sector hospitals in the Metro West region of Cape Town, South Africa
Source: BMC Infect Dis. 2023 Feb 3;23:67. doi: 10.1186/s12879-023-08027-z (PMC9896677; doi:10.1186/s12879-023-08027-z)
Supplement: Supplementary file 2 — Additional file 2: Table S2. Spectrum of bacterial isolates causing concomitant bacterial BSI and their antibiotic susceptibility patterns during Candida BSI. [file 12879_2023_8027_MOESM2_ESM.docx]

**Table S2**. Spectrum of bacterial isolates causing concomitant bacterial BSI and their antibiotic susceptibility patterns during *Candida* BSI

| **Bacteria isolated** | **Type of *Candida* bloodstream infection** | **Antibiotic susceptibility of Enterobacterales** | | |
| --- | --- | --- | --- | --- |
|  |  | 3rd generation cephalosporin-susceptible | 3rd generation cephalosporin-resistant | Carbapenem-resistant |
| *Klebsiella pneumoniae* (6) | *C. albicans* (4) | 0 | 4 | 0 |
|  | non-*C. albicans* (2) | 0 | 1 | 1 |
| *Enterobacter cloacae* (1) | *C. albicans* (0) | 0 | 0 | 0 |
|  | non-*C. albicans* (1) | 0 | 1 | 0 |
| *Escherichia coli* (1) | *C. albicans* (1) | 1 | 0 | 0 |
|  | non-*C. albicans* (0) | 0 | 0 | 0 |
|  | | **Antibiotic susceptibility of non-fermenter Gram-negative bacteria** | | |
|  |  | Beta-lactam-susceptible | Carbapenem-resistant | |
| *Pseudomonas aeruginosa* (3) | *C. albicans* (2) | 1 | 1 | |
|  | non-*C. albicans* (1) | 1 | 0 | |
| *Acinetobacter baumanii* (3) | *C. albicans* (2) | 1 | 1 | |
|  | non-*C. albicans* (1) | 0 | 1 | |
|  | | **Antibiotic susceptibility of *Staphylococcus aureus*** | | |
|  |  | Methicillin-susceptible | Methicillin-resistant but vancomycin-susceptible | |
| *Staphylococcus aueus* (5) | *C. albicans* (2) | 2 | 0 | |
|  | non-*C. albicans* (3) | 0 | 3 | |
|  | | **Antibiotic susceptibility of *Enterococcus faecium*** | | |
|  |  | Ampicillin-susceptible | Vancomycin-susceptible | |
| *Enterococcus faecium* (4) | *C. albicans* (1) | 0 | 1 | |
|  | non-*C. albicans* (3) | 0 | 3 | |
